# Supplementary material for: Clinical Application of Next-Generation Sequencing of Plasma Cell-Free DNA for Genotyping Untreated Advanced Non-Small Cell Lung Cancer
Source: Cancers (Basel). 2021 May 30;13(11):2707. doi: 10.3390/cancers13112707 (PMC8199488; doi:10.3390/cancers13112707)
Supplement: Supplementary file 1 [file cancers-13-02707-s001.zip › cancers-1186009-supplementary.pdf]

cancers

Supplementary files

Clinical Application of Next-Generation Sequencing of Plasma Cell-free DNA for Genotyping Untreated Advanced Non-Small Cell Lung Cancer

Maria Gabriela O. Fernandes, Natália Cruz-Martins, Conceição Souto Moura, Susana Guimarães, Joana Pereira Reis, Ana Justino, Maria João Pina, Adriana Magalhães, Henrique Queiroga, José Carlos Machado, Venceslau Hespanhol and José Luis Costa

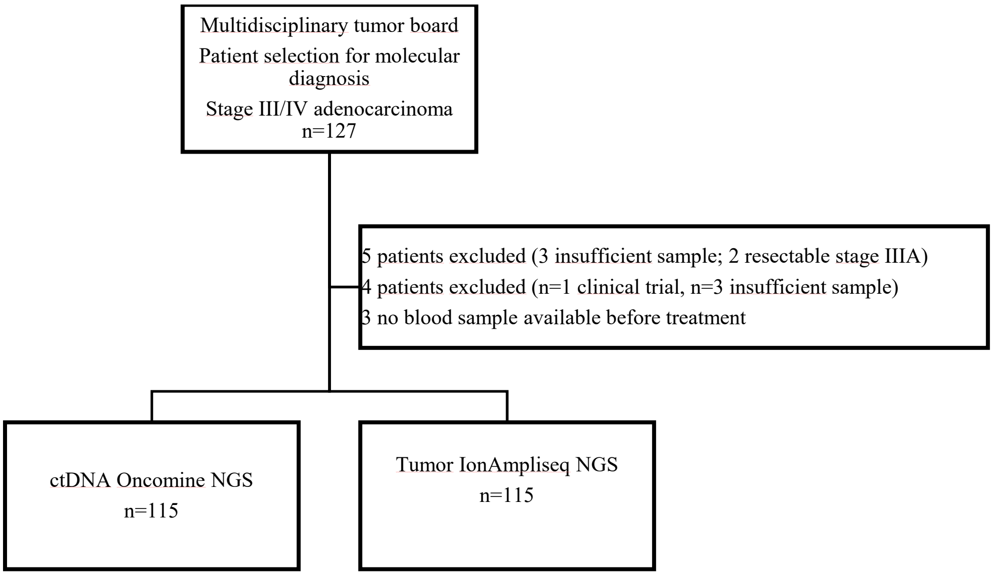

Figure S1. Study Design (ctDNA, circulating tumor DNA; NGS, Next-Generation sequencing) Of 127 patients with unresectable and stage IV lung adenocarcinoma, 115 had matched tumor and blood samples available for NGS testing at diagnosis.

Supplementary Data 1

Plasma Genotyping Tchnical Information.

Blood sampling, Cell-free DNA extraction and quantification

Sampling was obtained before patients had received any cancer treatment. Blood samples were collected in K2EDTA BD Vacutainer® PPT™ Plasma Preparation Tube (Becton Dickinson, Franklin Lakes, USA) and shipped at room temperature to the molecular laboratory in a time-lapse less than 4 h and plasma was immediately isolated upon receipt by centrifugation. Blood samples were centrifuged for 10 min at 1200 rpm, and the plasma supernatant was centrifuged for 10 min at 3000 rpm, then stored at -80C. Cell free DNA was extracted from aliquots (1 mL) of plasma with the MagMax Cell-Free Total Nucleic Acid Isolation Kit (Thermo Fisher Scientific, Waltham, USA) and quantified with the dsDNA HS assay kit by Qubit 3.0 or 4.0 Fluorometer (Thermo Fisher Scientific, Waltham, MA) following the manufacturer's instructions.

Library Preparation

Targeted-plasma NGS was performed using a validated amplicon-based NGS Oncomine™ Lung cfDNA Assay (Thermo Fisher Scientific, Waltham, MA), that uses target gene enrichment by PCR with a set of primers for exons of selected genes, covering more

than 150 hotspots on eleven genes, *ALK, BRAF, EGFR, ERBB2, KRAS, MAP2K1, MET, NRAS, PIK3CA, ROS1,* and *TP53*.

Next-Generation Sequencing, Variant Calling, and Bioinformatic Analysis

The libraries were sequenced with either the Ion PGM or Ion S5XL sequencer using semiconductor sequencing technology, following the manufacturer's instructions. The sequencing reads were aligned to the human reference hg19 genome with the Ion Torrent Suite V3.4.2 (or Torrent Suite Software™ v5.8). Accepted metrics for each sample were: number of reads per sample >2'500'000 reads (for Oncomine™ Lung cfDNA Assay libraries), on-target reads > 90%, read uniformity > 90%, mean depth> 20'000x. Coverage metrics for each amplicon was obtained by running the Coverage Analysis Plugin software v5.6.1 and later (Thermo Fisher Scientific).

Polymorphisms, synonymous or intronic mutations were excluded. The Catalogue of Somatic Mutations in Cancer (COSMIC) database was used to access the clinically relevant variants.

Supplementary Data 2

Tissue Genotyping Technical Information.

Tissue tumoral sampling, DNA extraction and quantification

Biopsy and cytology specimens, from the primary tumor and metastatic sites, were obtained at the time of first diagnosis and inspected through examination of hematoxylin and eosin-stained slides by a pathologist. Histology specimens were fixed in formalin (formalin-fixed paraffin-embedded, FFPE) and cytology specimens as smears and cellblocks. For DNA extraction, 4-5 FFPE tissue sections of 10 μm thickness, with at least 10% of tumor content, were cut and deparaffinized using Xylol. DNA extraction from tissue was performed using the QIAamp DNA Mini Kit (Qiagen, Hilden, Germany), following manufacturer´s instructions. DNA was quantified with NanoDrop Lite Spectrophotometer (Thermo Fisher Scientific., Waltham, MA, USA) or Qubit® 2.0 Fluorometer (Invitrogen, Waltham, MA, USA).

Library and Template Preparation for Next-Generation Sequencing

The Ion AmpliSeq Colon and Lung Cancer Research Panel v2 (Ion Torrent, Waltham, MA, USA) was used to detect DNA changes. This multiplex PCR-based test allows the analysis of 1850 hotspots and targeted regions in 22 genes (*AKT1, ALK, BRAF, CTNNB1, DDR2, EGFR, ERBB2, ERBB4, FBX7, FGFR3, FGFR1, FGFR2, KRAS, MAP2K1, MET, NOTCH1, NRAS, PTEN, PIK3CA, STK11, SMAD4* and *TP53)* involved in tumorigenesis. Libraries were generated using 1–10 ng of DNA from tissue FFPE blocks sections, according to the manufacturer.

Next-Generation Sequencing and Bioinformatic Analysis

Loaded chips were sequenced in an Ion PGM or Ion S5XL sequencer. Sequencing quality was assessed through the plugin coverage analysis, and the samples were analyzed using dedicated bioinformatic workflows within the Ion Reporter v5.6 server (Ion Torrent, Waltham, MA, USA). Samples with a number of reads <100,000 and/or the average base coverage <500_ were considered inadequate for analysis. The amplicons with a coverage <250_ were considered non-informative. Mutations with allele frequencies of at least 10% and adequate coverage in target regions were considered to call a mutation in a patient sample. Polymorphisms, synonymous or intronic mutations were excluded. The Catalogue of Somatic Mutations in Cancer (COSMIC) database was used to access the clinically relevant variants.

Supplementary data 3

Digital PCR Technique

TaqMan Mutation Detection Assays (Thermo Fisher Scientific) were used on a Quantstudio 3D digital PCR system (Thermo Fisher Scientific) to confirm discordant results between tumour and cfDNA results. The sensitivity for variant detection, according to the manufacturer, is down to 0.1%.

Supplementary data 4

Tissue Sample Types

Copyright: © 2021 by the authors. Licensee MDPI, Basel, Switzerland. This article is an open access article distributed under the terms and conditions of the Creative Commons Attribution (CC BY) license (http://creativecommons.org/licenses/by/4.0/).

**Title**	**Title**	**Tumor sample n**	**n (%)**
**Tumor specimen type**	Histologic	Lung 50<br>Bronchial 29<br>Pleura 10<br>Brain 2<br>Bone 2	93 (83.9)
	Cytologic	Pleural fluid 12<br>Lung-FNA 3<br>EBUS-FNA 2<br>Lymph node 4<br>Pericardial fluid 1	22 (19.1)

EBUS-FNA-endobronchial ultrasound- fine needle aspiration; FNA-fine needle aspiration.

Supplementary data 5

Evaluation of Factors Determining ctDNA Positivity

Title	**Plasma-positive**	**Plasma-negative**	**P value**
Predominant Location, n (%)<br>Lower lobes<br>Upper lobes<br>Bilateral<br>Hemithorax	 18 (43.9)<br>17 (41.5)<br>3 (7.3)<br>3 (7.3)	 28 (38.9)<br>35 (48.6)<br>6 (8.3)<br>3 (4.2)	0.808
Size (T), median (min-max)	47.0 (11.0-147.0)	40.5 (15.0-112.0)	0.417

Size (N), median (min-max)	14.0 (0-78.0)	13.0 (0-60.0)	0.381
Tumor volume* (mm^3), median (min-max)	21203.0 (445-556321.5)	19654.6 (648-475154.5)	0.879
Type of sample, n (%)			
Cytologic	7 (16.7)	15 (20.5)	0.610
Histologic	35 (83.3)	58 (79.5)	

* volume was determined using the following formulae: v (mm^3) = (length × $width^2$)/ 2, where length represents the largest tumor diameter and width represent the perpendicular tumor diameter.
